# Precision of CT-based micromotion analysis is comparable to radiostereometry for early migration measurements in cemented acetabular cups

**DOI:** 10.1080/17453674.2021.1906082

**Published:** 2021-04-06

**Authors:** Cyrus Brodén, Olof Sandberg, Henrik Olivecrona, Roger Emery, Olof Sköldenberg

**Affiliations:** a Department of Surgery and Cancer, Imperial College London, St Mary’s Hospital, London, UK; b Department of Clinical Sciences, Karolinska Institutet, Danderyd Hospital, Division of Orthopaedics, Stockholm, Sweden; c Sectra, Linköping, Sweden; d Department of Molecular Medicine and Surgery, Karolinska Institutet, Stockholm, Sweden

## Abstract

Background and purpose — CT (computed tomography) based methods have lately been considered an alternative to radiostereometry (RSA) for assessing early implant migration. However, no study has directly compared the 2 methods in a clinical setting. We estimated the precision and effective radiation dose of a CT-based method and compared it with marker-based RSA in 10 patients with hip arthroplasty.

Patients and methods — We included 10 patients who underwent total hip replacement with a cemented cup. CT and RSA double examinations were performed postoperatively, and precision and effective dose data were compared. The CT data was analyzed with CT micromotion analysis (CTMA) software both with and without the use of bone markers. The RSA images were analyzed with RSA software with the use of bone markers.

Results — The precision of CTMA with bone markers was 0.10–0.16 mm in translation and 0.31°–0.37° in rotation. Without bone markers, the precision of CTMA was 0.10–0.16 mm in translation and 0.21°–0.31° in rotation. In comparison, the precision of RSA was 0.09–0.26 mm and 0.43°–1.69°. The mean CTMA and RSA effective dose was estimated at 0.2 mSv and 0.04 mSv, respectively.

Interpretation — CTMA, with and without the use of bone markers, had a comparable precision to RSA. CT radiation doses were slightly higher than RSA doses but still at a considerably low effective dose.

Early migration of hip implants is associated with higher revision rates of prosthesis due to aseptic loosening (Kärrholm et al. 1994, Pijls et al. 2012). Radiostereometry (RSA) is the current gold-standard method to measure implant migration, given its accuracy and precision (Valstar et al. 2005). Lately there has been greater interest in using CT scans to measure implant migration to address some of the challenges with RSA, such as the need for specialized equipment and trained personnel to conduct and analyze examinations (Brodén et al. 2016, Otten et al. 2017). Previous experimental and clinical studies indicate that the accuracy and precision of CT techniques are comparable to those of RSA (Brodén et al. 2016, Scheerlinck et al. 2016, Brodén et al. 2019, 2020). However, to our knowledge there is no clinical study directly comparing CT and RSA in terms of precision for migration measurements on the same subjects. Recently a new commercial CT-based method, CT micromotion analysis (CTMA), was developed to analyze and measure implant migration between 2 CT images (Brodén et al. 2019, 2020). CTMA has features that make it possible to perform the migration analysis of CT data with tantalum beads and also with a technique relying solely on the surface anatomy of bone for the image registration, without the use of beads in the bone (Brodén et al. 2020). We compared the precision and effective dose of the 2 methods of CTMA with those of standard marker-based RSA in acetabular cups in patients with total hip arthroplasty (THA).

## Patients and methods

### Study setup

We selected the last 10 patients (mean age 67 [59–75]; 6 female; 10 hips) from a randomized study at the Orthopaedic Department of Danderyd Hospital comparing proximal migration of 2 types of cemented cups: an argon gas–sterilized polyethylene (PE) group and a Vitamin E–treated PE group (Muller Exceed ABT, Biomet, Warsaw, IN, USA). Included in that study were patients aged 40–75 years who had undergone THA (Sköldenberg et al. 2016).

A posterior approach was used for the surgical procedure. The femoral component of the surgery consisted of an uncemented tapered, proximally porous-coated and hydroxyapatite-coated stem composed of a Ti-6Al-4V titanium alloy (Bi-Metric HA, Biomet) and a 32-mm chromium–cobalt head.

Perioperatively, tantalum beads were inserted in the pelvis and liner of the cup. Patients were immediately mobilized at full weight-bearing with walking aids. The registration number of this clinical trial is NCT02254980.

### Data collection

Follow-up for each patient involved a double examination at 1 week or at 3 months postoperatively. 10 double examinations were performed according to the following procedures: (1) positioning the patient in an RSA calibration cage, (2) taking the RSA radiographs, (3) repositioning the X-ray tubes, calibration cage and patient on the table, (4) taking an additional set of RSA radiographs, (5) moving the patient to a CT scanner, (6) taking 1 CT scan, (7) repositioning the patient in the CT scanner, and (8) taking an additional CT scan.

### RSA method

For the RSA technique, we used a uniplanar calibration cage (Cage 43, RSA Biomedical AB, Umea, Sweden). Digital radiographs (Bucky Diagnost Philips, Eindhoven, the Netherlands) were taken using 2 X-ray sources angled at 40° to each other. The exposure was set to 120 kV and 8 mAs for each X-ray tube. UmRSA 6.0 computer software (RSA Biomedical, Umea, Sweden) was used for all the RSA migration analyses. The condition numbers (the spread of the markers) were below 100, and all mean errors of rigid body fitting (the stability of the markers) were below 0.30 mm.

### CT method

We used a CT scanner (Discovery CT750HD, GE Healthcare, Chicago, IL, USA) to acquire CT scans. The CT protocol was set to 120 kV, 10 mAs, slice thickness 0.625 mm with an increment of 0.31 mm, rotation time 0.6 s, pitch 0.894. Volumes were reconstructed with an x–y–z resolution of 0.6–0.6–0.6 mm.

**Figure 1.**
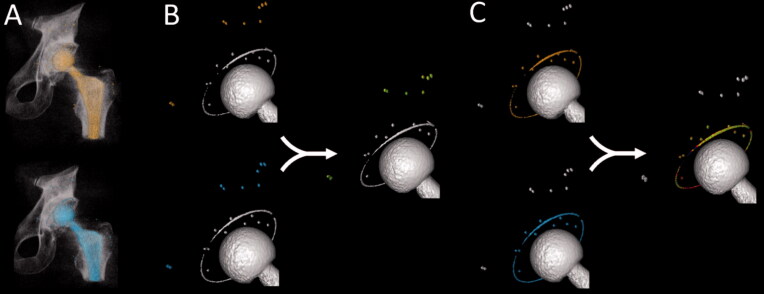
CTMA analysis workflow, using beads inserted in the bone for pelvic bone image registration. (1) First, a segmentation threshold of 2200 Hounsfield units is set to visualize the metallic structures in the pelvic bone (orange and blue). (2) The beads in the pelvic bone are selected. (3) When the registration occurs, green indicates a 1st successful registration. (4) A segmentation threshold of 2200 Hounsfield units is set to visualize the metallic structures of the cup, such as beads and threads in 2 separate CT images. The beads and the thread of the cup are selected. (5) Registration occurs; green indicates a successful 2nd registration.

The CT scans were analyzed with the image registration software CTMA (Sectra, Linköping, Sweden). CTMA makes it possible to analyze migration between 2 rigid bodies, such as the cup and the bone, in between 2 CT examinations (see Figure 1 for steps performance) (Brodén et al. 2020). In this study, the following steps were performed.

1. We imported 2 CT volumes into the CTMA software. A threshold segmentation for beads in the bone (2200 Hounsfield units [HU]) or bone thresholding (600 HU) was set. The segmentation allowed us to visualize the beads or bone depending on the CTMA technique that was selected. The same bone threshold was used for all examinations except in 2 patients, where the threshold was set to 400 HU and 550 HU due to deviances in the CT reconstruction settings.
2. The beads or the surface anatomy of the pelvis was selected for the image registration of the pelvic bone.
3. The pelvic bone was registered, and a visual overlap of the pelvic beads or the surface of the pelvic bone was achieved.
4. A threshold segmentation of 2200 HU was set to visualize the metallic implant structures such as the thread and beads of the cup. The thread and beads were selected for the image registration of the implant.
5. The implant was registered, and a visual overlap of the beads and thread of the implant was achieved.
6. The software calculated the motion of the center of mass of the implant compared with bone between these 2 CT volumes in a CT-based coordinate system. The result was a visual output in the form of registered 3D volumes and numerical migration values expressed in six degrees of freedom (translation along and rotation around x–y–z axis in a CT Dicom coordinate system).
7. The CT coordinate system was thereafter modified in a multi-planar reconstruction (MPR) view to obtain a coordinate system comparable to the RSA coordinate system.

The CTMA procedure described in steps 1–7 was performed once using the beads in the bone and once using the surface pelvic anatomy (without beads in the bone) for steps 1–3.

This CTMA procedure has previously been illustrated and described (Brodén et al. 2019, 2020).

### CT and RSA coordinate systems

**Figure 2.**
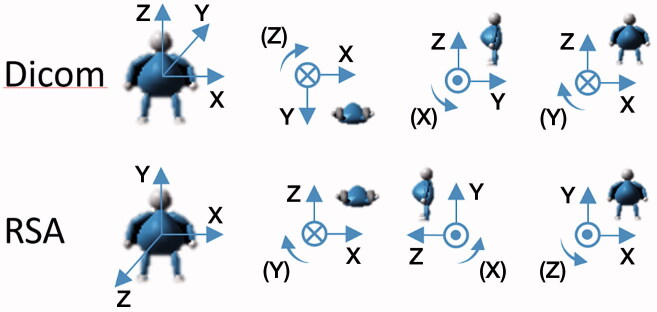
CT Dicom coordinate system of CTMA and coordinate system of RSA. The translations are positive in the direction of the arrow, and the rotations are positive in a clockwise direction.

The coordinate systems used in RSA and CTMA differ (Figure 2). The RSA coordinate system is anatomical, fixed, and defined by the RSA calibration cage. The CTMA used the standard Dicom coordinate system that could be modified into that of the RSA coordinate system.

### Effective radiation dose

The CT effective dose was estimated using the dose length product (DLP) multiplied by a pelvic conversion factor: 0.0129 mSv/mG.cm from IRCP 103, but also with a Monte Carlo simulation in an Impact CT dosimeter software (Deak et al. 2010, Saltybaeva et al. 2014).

The RSA effective dose was estimated with a Monte Carlo simulation using a software PCXMC Dose Calculation available at Danderyd Hospital (STUK, Helsinki, Finland version 2.0.1.4) (Tapiovaara and Siiskonen 2008).

### Precision

Precision is defined by the proximity between repeated measurements under similar conditions (ISO 16087:2013 2013). According to this standard, “Precision should be assessed with double measurement and shall be presented as the standard deviation of these calculated migrations. Assuming a normal distribution, the confidence intervals of the error should be expressed as ± 1,96 × SD, for the 95% confidence interval (where SD is the standard deviation).”

### Statistics

For the precision calculation, we used the ISO standard for the 95% confidence interval (CI) 1.96 x SD, but modified to the formula T-score x SD to take into account the small sample size, a practice prevalent in previous studies using this method (Sköldenberg and Odquist 2011, Brodén et al. 2020). The T-score was used instead of a Z-score to avoid underestimating the margin of error obtained with this small sample size.

### Ethics, data sharing plan, funding, and conflicts of interests

The Ethical Committee of the Karolinska Institute approved the use of CT scans in this study (No. 2011/2003-31/1).

The clinical data of this study will be available upon request at: cyrus.broden@gmail.com. OSK and RE did not have any conflict of interests. CB and HO received consultancy fees from Sectra Orthopaedics. OSA is a full-time employee at Sectra. Sectra Orthopaedics had no involvement in the data collection, analysis or interpretation of the data.

## Results

The precision of CTMA using tantalum beads inserted in the bone for the pelvic bone registration varied between 0.10 and 0.16 mm in translation and 0.31° and 0.37° in rotation (Table 1, Table 2 Supplementary data). All the implanted beads in the bone and the implant itself were visualized.

The precision of CTMA using bone surface anatomy for the bone registration varied between 0.10 and 0.16 mm in translation and 0.21° and 0.31° in rotation (Table 1, Table 3 Supplementary data). Some artefacts could be observed at the surface of the pelvic bone, but no patients needed to be excluded.

The precision of the gold-standard RSA for this cohort varied between 0.09 and 0.26 mm in translation and 0.43° and 1.69° in rotation (Table 1, Table 4 Supplementary data). One patient was excluded from the RSA measurements due to marker occlusion.

The CT mean effective radiation dose of the scans used in the CTMA analysis was estimated to be 0.2 mSv (0.10–0.22) with both techniques of dose calculation. The RSA mean effective dose was estimated to be 0.04 mSv (0.036–0.044).

## Discussion

The CTMA method was more precise in terms of rotation compared with RSA. We speculate this is because CT images made additional surfaces available compared with RSA for the image analysis, such as the surface anatomy of the pelvis, metallic thread of the cups, and additional markers since marker occlusion did not occur. This created a more rotational stable rigid body and improved the precision of our registrations in CTMA. These results are in accordance with a clinical study of another CT-based method conducted by Otten et al. (2017) where the limit of agreement between the CT and RSA varied between 1.05° and 2.17° in rotation. However, in our study CTMA using beads and surface anatomy was as precise as standard RSA in translation. These results were below the critical threshold of 1 mm of early migrations that is suggested to predict loosening and could therefore be used in a clinical setting (Pijls et al. 2012).

The precision of RSA for this cohort varied between 0.09 and 0.26 mm in translation and 0.43° and 1.69° in rotation. This is in accordance with a review paper by Kärrholm (2012) indicating that RSA precision in clinical studies varied between 0.15 and 0.60 mm for translations and between 0.3° and 2° for rotation. These values are less precise than those of RSA in an experimental setting, especially in rotation, ranging from 0.04 to 0.09 mm for translations and 0.08° to 0.32° for rotations (Brodén et al. 2016). This difference might be due to the use of sawbones without soft tissues in the experimental study by Brodén et al.

The CTMA software without beads includes only common anatomical structures between 2 CT volumes for image registration. Therefore, anatomical changes such as ectopic bone formation, cortical thickening, or osteolysis, which might occur over a longer study time, might possibly not influence the precision of the method. However, with methods relying on beads inserted in the bone, bone remodelling over time might affect the positioning of the beads, which could affect the extrapolation of postoperative precision data to other timepoints.

The CT effective dose was estimated using the dose length product (DLP) multiplied by a pelvic conversion, the most commonly used method in clinical routine (Deak et al. 2010). However, we also estimated the effective dose with a Monte Carlo simulation in Impact CT dosimeter software, since it is considered the most accurate effective dose-estimation method, due to its ability to model the interaction between radiation and matter, considering several parameters of the CT protocol (Saltybaeva et al. 2014). The mean effective dose of CTMA with both methods was 0.2 mSv, while the clinical RSA effective dose was 0.04 mSv. In a study by Blom et al. (2020), the effective dose of RSA was estimated to be 0.04 mSv, similar to our findings. The complexity of acquiring RSA images where patient and calibration cage are lined up correctly means that some additional retakes of RSA images could increase the effective dose for RSA. Retakes were not included in our calculation of RSA effective dose. The effective dose for CT is 5 times higher than for RSA in our study. However, it is lower than a standard anteroposterior pelvic radiograph (0.7 mSv) (Boettner et al. 2016). Moreover, the practicalities of CT, in addition to the potential to visualize the bone–implant interface, might justify the slightly higher dose. It is important to consider that this CT dose is suboptimal, because artefacts were observed, and higher doses could theoretically facilitate the analysis of CT scans with CTMA relying solely on the surface anatomy of the bone. The CT dose in this study is markedly at the lower end compared with those used in other CTMA studies with an effective dose ranging between 0.2 and 2.3 mSv (Brodén et al. 2020 ). However, CTMA precision does not appear to have been markedly affected by this lower dosage.

An advantage of CTMA technology is the wide availability of CT scanners in hospitals, which facilitates early migration measurement of implants without the need for expensive investments in RSA laboratories. For RSA, imaging acquisition must be carefully monitored by a specially trained radiographer. For CTMA, a predetermined CT-scan protocol is chosen by a radiographer for the scan, and the positioning of the patient is not as crucial as in RSA, as no calibration cage is involved.

Another advantage of CTMA is that the registration is performed in a 3D visual interface, which prevents the loss of tantalum markers due to marker occlusion. In our study, 1 RSA examination had to be excluded due to marker occlusions, which did not occur with CTMA.

A limitation of this study is the small sample size. No statistical test was performed due to the small sample size.

A limitation of the CTMA software is that the quality of the image registration must be verified manually. This is done in a 1st step with a visual colourmap feedback mechanism. In a 2nd step, a visual inspection in 2D of the axial, frontal, and sagittal view of the CT images is performed after each registration to verify if the bone or implant is well registered. Currently in CTMA, there is no equivalent number to the condition numbers and mean error of rigid body fitting to quantify the suitability of the rigid body to give correct measurements; this assessment must rely on the user’s experience and judgment. The CTMA analyses were performed by an experienced CTMA user (CB). Although Sandberg et al. (2020) previously have shown that CTMA precision can be reproduced by an inexperienced user, it is important to emphasize the importance of CTMA training before the use of this tool.

We did not investigate the precision of CTMA for the femoral stem. For future investigations, a slightly higher effective dose might be needed to analyze the femur/stem migration. The stem is a metallic component, and the presence of soft tissue around the femur might increase the effective dose needed to obtain CT images of adequate quality without artefacts. Moreover, the whole stem was not included in the field of view of the CT scans, which could impact the ability to measure stem precision.

In conclusion, we found that CTMA, with and without the use of bone markers for image registration, had a comparable precision when compared with standard RSA, and a slightly higher effective dose for cups in hip arthroplasty while still at a considerably low effective dose.

## Supplementary Material

Supplemental Material

## Supplementary data

Tables 2–4 are available as supplementary data in the online version of this article, http://dx.doi.org/10.1080/17453674.2021.1906082
