# Supplementary material for: Precision of CT-based micromotion analysis is comparable to radiostereometry for early migration measurements in cemented acetabular cups
Source: Acta Orthop. 2021 Apr 6;92(4):419–23. doi: 10.1080/17453674.2021.1906082 (PMC8381926; doi:10.1080/17453674.2021.1906082)
Supplement: Supplemental Material [file IORT_A_1906082_SM2295.pdf]

Supplementary data

Table 2. Precision of CTMA of the cup using beads in the bone for the pelvic bone registration

	Precision	Registration errors			
	n = 10	mean	minimum	maximum	SD
X translation, mm	0.14	0.02	–0.05	0.17	0.06
Y translation, mm	0.10	0.02	–0.07	0.10	0.04
Z translation, mm	0.16	0.01	–0.12	0.13	0.07
X rotation, °	0.31	0.03	–0.14	0.31	0.14
Y rotation, °	0.37	–0.04	–0.31	0.22	0.16
Z rotation, °	0.33	–0.03	–0.32	0.20	0.15

SD = standard deviation

Table 3. Precision of CTMA of the cup using pelvic surface bone anatomy for the bone registration

	Precision	Registration errors			
	n = 10	mean	minimum	maximum	SD
X translation, mm	0.16	0.02	–0.03	0.20	0.07
Y translation, mm	0.14	0.01	–0.05	0.09	0.06
Z translation, mm	0.10	0.00	–0.07	0.09	0.04
X rotation, °	0.25	–0.04	–0.28	0.12	0.11
Y rotation, °	0.21	–0.11	–0.23	0.10	0.09
Z rotation, °	0.31	0.00	–0.24	0.19	0.14

SD = standard deviation

Table 4. Precision of RSA of the cup using beads

	Precision	Registration errors			
	n = 9	mean	minimum	maximum	SD
X translation, mm	0.09	0.01	–0.05	0.08	0.04
X translation, mm	0.20	0.01	–0.10	0.20	0.09
X translation, mm	0.26	–0.06	–0.26	0.07	0.11
X rotation, °	1.69	–0.05	–0.89	1.60	0.75
Y rotation, °	1.56	–0.27	–1.97	0.26	0.69
Z rotation, °	0.43	0.12	–0.16	0.36	0.19

SD = standard deviation
